# Not All Thwarting Is Equal: Distinct Temporal Patterns Linking Teachers' Psychological Needs to Turnover Intention

**DOI:** 10.1002/brb3.71861

**Published:** 2026-10-28

**Authors:** Qi Song, Xiao‐Yan Zou, Cong Liu, Shan Xu, I‐Hua Chen

**Affiliations:** ^1^ Faculty of Education Qufu Normal University Qufu China; ^2^ Department of Life Sciences Changzhi University Changzhi China; ^3^ School of Business Zibo Polytechnic University Zibo China; ^4^ School of Computer and Information Engineering Dezhou University Dezhou China; ^5^ Faculty of Education Yunnan Normal University Yunnan China; ^6^ School of Artificial Intelligence Hubei Preschool Teachers College Wuhan China; ^7^ Chinese Academy of Education Big Data, Faculty of Education Qufu Normal University Qufu China

**Keywords:** Chinese teachers, longitudinal study, psychological need thwarting, turnover intention

## Abstract

**Background:**

Teacher turnover intention has become a critical global concern, compromising teachers' psychological well‐being, school workforce stability, and student learning outcomes. While psychological need thwarting (PNT) is recognized as a key antecedent of turnover intention, existing research has predominantly treated PNT as a unidimensional construct and overlooked the possibility that turnover intention may reciprocally shape teachers' subsequent need experiences. Integrating self‐determination theory and conservation of resources theory, this study employed a two‐wave cross‐lagged panel model to examine bidirectional associations between the three PNT dimensions and turnover intention among Chinese teachers.

**Methods:**

Data were collected from 1,061 teachers at Time 1 and 812 at Time 2 under a design permitting voluntary, wave‐specific participation, with 332 teachers participating in both waves (*M* age = 41.19 years, *SD* = 9.13; 69.6% female); cross‐lagged models controlling for gender and age were estimated using full information maximum likelihood, drawing on all available observations from 1,541 unique participants, with school ownership and school type incorporated into the missing‐data model.

**Results:**

An asymmetric temporal pattern emerged: among the three dimensions, only autonomy thwarting predicted subsequent turnover intention (B = 0.09, 95% CI [0.01, 0.17], β = 0.12, *p* = 0.023), whereas turnover intention predicted later competence thwarting (B = 0.22, 95% CI [0.02, 0.41], β = 0.11, *p* = 0.031) and relatedness thwarting (B = 0.19, 95% CI [0.01, 0.37], β = 0.13, *p* = 0.048) but not autonomy thwarting (B = 0.08, 95% CI [−0.07, 0.24], β = 0.06, *p* = 0.276). Although these cross‐lagged effects were small in magnitude, they demonstrated distinct directional patterns across the three needs.

**Conclusion:**

These findings suggest that turnover intention may function not only as an outcome of adverse need experiences but also as an antecedent of subsequent need states. This asymmetric pattern points to potentially relevant intervention targets: autonomy support for teachers who have not yet developed turnover intention, and competence and relatedness support for those already considering leaving.

## Graphical abstract

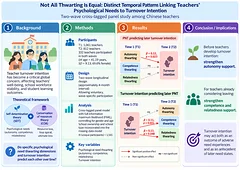

## 1 Introduction

In recent years, work‐related challenges have intensified turnover intention among teachers (OECD 2020), a phenomenon recognized as a pressing concern internationally (e.g., Mäkelä and Whipp 2015; McInerney et al. 2015; Skaalvik and Skaalvik 2011, 2017). Turnover intention of teachers refers to a conscious desire to leave one's current educational institution or to exit the teaching profession entirely, and is widely regarded as an adverse work outcome often emerging from unmet psychological needs (McInerney et al. 2015; Shim et al. 2022). According to self‐determination theory (SDT; Deci and Ryan 2000), psychological need thwarting (PNT), defined as the frustration of needs for autonomy, competence, and relatedness, is associated with diminished performance and ill‐being, such as turnover intention (Gillet et al. 2015a; Olafsen and Deci 2020). However, most studies have conceptualized PNT as a unitary antecedent of turnover intention, treating the latter solely as a downstream outcome, and have examined PNT as a global construct rather than attending to its distinct dimensions (Gillet et al. 2015a; Huyghebaert et al. 2018; Olafsen et al. 2017). Conservation of resources (COR) theory (Hobfoll et al. 2018) offers a complementary perspective by highlighting the possibility of a reverse temporal association: heightened turnover intention may reflect a state of resource strain that, through defensive resource‐conserving responses, could be linked to subsequent increases in PNT. Moreover, distinct dimensions of PNT may play differential roles in this dynamic process. Because empirical research has yet to synthesize these perspectives, the present study adopts an integrated SDT‐COR framework to examine the bidirectional temporal associations between the three dimensions of PNT and turnover intention over time.

### 1.1 Self‐Determination Theory: From Psychological Need Thwarting to Turnover Intention

SDT provides a comprehensive framework for understanding motivation, engagement, and functioning in various life domains (Gagné and Deci 2005). Central to SDT is basic psychological needs theory, which posits that autonomy, competence, and relatedness function as essential nutrients for psychological growth and integrity (Deci and Ryan 2000). When these needs are satisfied, optimal functioning is fostered; when they are thwarted, psychological integrity is undermined, contributing to maladaptive outcomes (Bartholomew et al. 2014; Vansteenkiste and Ryan 2013). Notably, PNT is conceptually distinct from mere need dissatisfaction, representing an active deprivation rather than simply the absence of fulfillment (Vansteenkiste and Ryan 2013; Vansteenkiste et al. 2020).

In the teaching context, the three dimensions of PNT manifest distinctly. Autonomy thwarting involves perceptions of external control and pressure (Bartholomew et al. 2011), occurring when teachers encounter rigid instructional constraints or are excluded from decision‐making. Competence thwarting refers to feelings of inefficacy and inadequacy (Bartholomew et al. 2011; Van den Broeck et al. 2016), typically triggered by insufficient resources, unattainable demands, or harsh criticism. Relatedness thwarting is characterized by alienation and isolation (Bartholomew et al. 2011; Deci et al. 2017), arising when teachers perceive neglect, conflict, or hostility from colleagues, administrators, or students. Although conceptually distinct, these three dimensions are interrelated (Vansteenkiste et al. 2020). For instance, inability to implement instructional strategies autonomously may lead to poor student outcomes, initially triggering autonomy thwarting and subsequently inducing competence thwarting. Furthermore, teachers experiencing autonomy and competence thwarting may avoid interactions due to shame, thereby exacerbating relatedness thwarting.

According to SDT, PNT reflects adverse experiences that threaten psychological growth and well‐being, prompting self‐protective and defensive psychological readjustments (Deci and Ryan 2000). Such readjustments may manifest as withdrawal from one's current position and intentions to seek alternative employment opportunities, namely turnover intention. Empirical research in occupational settings has consistently supported this proposition. Global need thwarting has been found to exert both direct and indirect effects (via affective commitment) on turnover intentions among executives (Gillet et al. 2015a). Also in the enterprise context, Olafsen et al. (2017) identified a “dark path” of motivational processes whereby PNT was related to increased work stress, which in turn was associated with higher turnover intentions among employees. Besides, PNT has been identified as a mediator between psychosocial safety climate and turnover intention among nurses (Huyghebaert et al. 2018). Person‐centered studies have further corroborated these findings. For instance, Jungert et al. (2025) identified four distinct profiles of psychological need satisfaction, frustration, and unfulfillment among employees, revealing that membership in predominantly negative need states profiles was associated with higher turnover intentions, whereas membership in predominantly positive profiles was associated with lower turnover intentions. Beyond occupational contexts, comparable patterns have also emerged in educational settings, where need‐thwarting environments are associated with increased turnover intention among teachers (Collie 2023).

While this evidence is compelling, most studies have examined PNT as a unidimensional construct, potentially obscuring the distinct contributions of each thwarted need. Research examining the three dimensions separately has begun to illuminate their differential effects on teacher outcomes. In the context of teacher ill‐being, Bartholomew et al. (2014) demonstrated that each dimension of PNT independently mediated the relationship between job pressure and burnout among UK physical education teachers, with competence thwarting additionally predicting somatic complaints. Regarding turnover intention specifically, the limited available evidence suggests that the three dimensions may contribute differentially. Shim et al. (2022) found that among European American teachers, autonomy frustration was the strongest predictor of intention to leave, followed by relatedness frustration, whereas competence frustration showed no significant effect. Similarly, Collie (2023) reported that among Australian school teachers, relatedness with colleagues was negatively associated with turnover intentions, while autonomy‐thwarting leadership practices were positively associated with turnover intentions; competence‐related variables were not examined in that study. Person‐centered research offers a complementary perspective. Using latent profile analysis with Finnish teachers, Heinimäki et al. (2026) identified distinct need fulfillment profiles and found that teachers in the “Socially Unfulfilled” profile, characterized primarily by low relatedness satisfaction and high relatedness frustration, reported the highest turnover intentions, while those in the “Dominantly Fulfilled” profile reported the lowest. Notably, an informative contrast emerged between two intermediate profiles: teachers in the “Socially Fulfilled” profile, who reported high relatedness satisfaction alongside moderate to high autonomy and competence thwarting, exhibited higher turnover intention than those in the “Autonomy‐Oriented with Low Competence Frustration” profile, who reported moderately elevated autonomy satisfaction and notably low competence frustration. This pattern suggests that deficits in autonomy and competence may outweigh the protective effects of relational fulfillment. Although methodological differences across studies preclude direct comparison of effect sizes, converging evidence consistently highlights the differential roles of autonomy, competence, and relatedness in shaping teachers' turnover intentions. These findings underscore the necessity of examining the three dimensions separately rather than aggregating them into a global construct, as the relative salience of each need may vary across outcomes and contexts.

In summary, SDT and prior research have established PNT as a robust mechanism linking adverse work experiences to turnover intention. However, existing studies are predominantly cross‐sectional, leaving the temporal dynamics of this relationship unclear. Limited longitudinal evidence exists; for instance, one cross‐lagged study demonstrated that psychosocial safety climate was negatively related to need thwarting, which predicted increased burnout three months later; burnout in turn explained elevated turnover intention among employees (Huyghebaert et al. 2018). Nevertheless, systematic longitudinal evidence regarding the independent temporal pathways through which the three PNT dimensions relate to turnover intention remains lacking.

### 1.2 Conservation of Resources Theory: From Turnover Intention to Psychological Need Thwarting

While SDT provides a framework for understanding how PNT leads to turnover intention, COR theory (Hobfoll et al. 2018) offers a complementary perspective for theorizing the reverse pathway, namely, how turnover intention may be associated with later PNT. COR theory posits that individuals strive to protect and accumulate valued resources, and when resources are threatened or lost, stress ensues. Three core principles are particularly relevant: (a) the primacy of resource loss principle, which suggests that resource loss is disproportionately more salient than resource gain; (b) the loss spiral corollary, whereby initial resource loss begets further loss; and (c) the desperation principle, which states that individuals lacking resources become increasingly defensive and may engage in counterproductive behaviors to protect remaining resources. Applied to the present context, these principles suggest that teachers who develop turnover intention may enter a self‐reinforcing cycle in which their defensive responses could inadvertently deepen their experience of PNT.

Specifically, COR theory suggests that turnover intention may be accompanied by defensive withdrawal behaviors aimed at conserving remaining psychological resources (Hanisch and Hulin 1990; Kahn 1990). When teachers contemplate leaving, they may gradually distance themselves from students, colleagues, and institutional commitments, reducing emotional investment to protect themselves from further disappointment or resource drain. However, such withdrawal behaviors could inadvertently contribute to PNT across all three domains. Reduced engagement with students and colleagues may erode supportive relationships, deepening relatedness thwarting. Diminished investment in pedagogical improvement and professional development may accelerate feelings of inefficacy, exacerbating competence thwarting. In addition, psychological disengagement may be accompanied by fewer perceived opportunities for participation and control at work, which could contribute to autonomy thwarting. Thus, a self‐reinforcing loss spiral may emerge in which turnover intention and PNT mutually amplify one another over time. It should be noted, however, that these proposed mechanisms, such as defensive withdrawal and the erosion of workplace relationships, were not directly measured in the present study and are advanced here as theoretical propositions rather than tested processes.

Although no prior studies have directly examined whether turnover intention relates to subsequent PNT, two lines of indirect evidence provide a tentative rationale for considering this possibility. First, Lei et al. (2024) found in a three‐wave longitudinal study of early childhood teachers that higher turnover intention was associated with subsequent perceptions of a less favorable workplace climate. From the perspective of COR theory, such deterioration in perceived work environment quality may reflect a loss of organizational resources, which could constitute the initial step of a loss spiral in which further losses, including intensified PNT, may follow. Supporting this latter link, organizational resources have been shown to be negatively related to PNT (Gillet et al. 2015b), and low psychosocial safety climate, an unfavorable workplace climate offering insufficient protection of employees' psychological health and safety, has been associated with increased PNT (Huyghebaert et al. 2018). Second, longitudinal evidence suggests that teachers with persistent turnover intentions tend to experience higher levels of burnout (Räsänen et al. 2022), while related evidence from the sport psychology literature indicates that burnout may precede later increases in autonomy frustration (Shannon et al. 2022). Collectively, these findings suggest that turnover intention may be followed by deteriorated workplace climate and psychological strain, both of which could affect later need‐thwarting experiences. However, because this evidence remains indirect, the present study examined the reverse pathway from turnover intention to later PNT as an exploratory question and interpreted the findings cautiously.

In summary, whereas SDT provides the theoretical basis for understanding how PNT may be linked to later turnover intention, COR theory extends this perspective by suggesting the possibility that turnover intention may be associated with later PNT. Together, these complementary frameworks provide a tentative rationale for considering a bidirectional temporal relationship between PNT and turnover intention. Nevertheless, direct longitudinal tests of this reciprocal relationship remain absent from the literature, underscoring the need for empirical investigation using appropriate analytical approaches capable of modeling bidirectional temporal dynamics.

### 1.3 Present Study

Despite substantial evidence linking PNT to turnover intention, two notable gaps remain. First, existing research is predominantly cross‐sectional, leaving the temporal dynamics of this relationship unclear and precluding examination of potential reverse pathways. Second, prior studies have frequently treated PNT as a unitary construct, implicitly assuming that the three dimensions contribute equally to turnover intention, an assumption that is theoretically unwarranted given their conceptual distinctiveness. The present study addresses these gaps by examining the bidirectional temporal associations between the three dimensions of PNT and turnover intention among teachers using a two‐wave longitudinal design. Drawing on SDT, it is expected that autonomy thwarting, competence thwarting, and relatedness thwarting at Time 1 will each positively predict turnover intention at Time 2, though the strength of these associations may differ across dimensions (Hypothesis 1). Beyond this well‐established forward direction, the present study also considers the reverse pathway. Drawing on COR theory, particularly its loss spiral corollary, this pathway was examined as a theoretically informed but exploratory question: turnover intention at Time 1 was tentatively expected to positively predict autonomy thwarting, competence thwarting, and relatedness thwarting at Time 2, with potentially differential associations across dimensions (Hypothesis 2).

## 2 Methods

### 2.1 Procedures and Participants

To investigate the temporal association between teachers' PNT and turnover intention, a two‐wave longitudinal design with a four‐month interval and voluntary, wave‐specific participation (see below) was employed. Data were collected through online surveys at two time points: Time 1 (T1) in February 2024, coinciding with the beginning of the spring semester, and Time 2 (T2) in June 2024, at the semester's conclusion.

Participation in each wave was voluntary and non‐mandatory: teachers could take part at T1 only, at T2 only, or at both waves. This decision was made deliberately, for both methodological and ethical reasons. Given the sensitive nature of measuring turnover intention among active teachers, a traditional rigid follow‐up design requiring mandatory participation at both time points could raise concerns about surveillance, potentially compromising response honesty and inducing anxiety about job security. Such concerns might be particularly acute in educational settings where turnover intentions could be perceived as disloyalty or lack of commitment. Permitting voluntary participation at either or both measurement points addresses these concerns. Specifically, participants who completed the baseline survey could opt out of the follow‐up assessment, while those unable to participate at T1 could still contribute data at T2. This design strategy serves dual purposes: (1) it reduces the feeling of being “tracked” for potential turnover, thereby encouraging more candid responses about sensitive career intentions, and (2) it accommodates teachers' varying availability during high‐demand periods (e.g., examination preparation, grading periods), thereby minimizing systematic attrition of teachers experiencing the highest workloads and improving sample representativeness. It should be emphasized that this constitutes an accommodated rather than a planned missing data design (Graham et al. 2006; Little and Rhemtulla 2013): the pattern of wave participation was determined by participants rather than assigned by the researchers, and the resulting missingness therefore cannot be assumed to be missing completely at random (MCAR). The implications of this for the missing data mechanism and the estimation strategy are examined below.

Participants were recruited through convenience sampling via institutional channels, specifically through the research team's professional networks and collaborating local education bureaus. Upon obtaining institutional consent, school administrators from participating schools disseminated the online survey link to their teaching staff. Because recruitment relied on established institutional contacts, the resulting sample was geographically concentrated, with the majority of respondents (77.0% at T1 and 77.3% at T2) teaching in Jiangsu Province, an economically developed eastern coastal region of China; the remaining participants were drawn from other provinces. This geographic concentration is addressed further in the Limitations section. Inclusion criteria stipulated that participants must be: (a) currently employed as in‐service teachers, and (b) without any diagnosed clinical mental disorders. The latter criterion was verified through a self‐report screening item at the beginning of the survey asking whether participants had ever received a clinical diagnosis of a mental disorder; no clinical interview or medical record verification was conducted. Prior to data collection, all participants provided electronic informed consent and were assured of complete data confidentiality and their right to withdraw from the study at any time without consequence.

Several measures were implemented to safeguard participant confidentiality. Surveys were completed anonymously online, with no names, employee identifiers, or other personally identifying information collected. To link responses across the two waves, participants provided only the last four digits of their citizen identification number, which served solely as a pairing code for data matching and was insufficient to identify any individual. Data were accessible only to the research team and were analyzed and reported exclusively in aggregate form. School administrators who disseminated the survey link had no access to individual responses and were not informed of which teachers participated. The research protocol received full ethical approval from the Institutional Review Board of the Jiangxi Psychological Consultant Association (IRB Reference: JXSXL‐2024‐JA08). All procedures were conducted in strict accordance with established ethical guidelines for research involving human participants.

The survey yielded 1,061 and 812 teacher respondents at T1 and T2, respectively, of whom 332 participated in both waves (69.6% female, n = 231; *M* age = 41.19 years, *SD* = 9.13). Table 1 presents demographic characteristics across the T1 sample, the T2 sample, and the both‐wave subsample. It should be noted that, because wave participation was voluntary, the reduction across waves reflects both conventional attrition and design‐permitted single‐wave participation, including participants who entered the study only at T2.

**TABLE 1:** Demographic characteristics of participants across study waves.

	T1 (*n* = 1061)	T2 (*n* = 812)	*Both Waves (n =* 332)
Gender: n (%)			
Male	329 (31.0%)	261 (32.1%)	101 (30.4%)
Female	732 (69.0%)	551 (67.9%)	231 (69.6%)
Age of Years: mean (*SD*)	40.33 (9.32)	41.09 (9.25)	41.19 (9.13)
Teaching experience of Years: n (%)			
5 years and under	225 (21.2%)	134 (16.5%)	55 (16.6%)
6–10 years	144 (13.6%)	136 (16.7%)	46 (13.9%)
11–15 years	97 (9.1%)	69 (8.5%)	31 (9.3%)
16–20 years	105 (9.9%)	65 (8.0%)	31 (9.3%)
21 years and above	490 (46.2%)	408 (50.2%)	169 (50.9%)
School ownership: n (%)			
Public	1017 (95.9%)	792 (97.5%)	329 (99.1%)
Private	44 (4.1%)	20 (2.5%)	3 (0.9%)
School type: n (%)			
Senior high school	72 (6.8%)	28 (3.4%)	4 (1.2%)
Senior vocational school	46 (4.3%)	43 (5.3%)	8 (2.4%)
Junior high school	271 (25.5%)	151 (18.6%)	75 (22.6%)
Junior vocational school	304 (28.7%)	264 (32.5%)	136 (41.0%)
Primary school	368 (34.7%)	326 (40.1%)	109 (32.8%)
Teaching subject: n (%)			
Chinese	269 (25.4%)	215 (26.5%)	95 (28.6%)
Mathematics	195 (18.4%)	154 (19.0%)	56 (16.9%)
English	132 (12.4%)	89 (11.0%)	38 (11.4%)
Professional subject (e.g., vocational subjects)	254 (23.9%)	196 (24.1%)	85 (25.6%)
Others	211 (19.9%)	158 (19.4%)	58 (17.5%)

*Note*. T1 = Time 1; T2 = Time 2; *SD* = standard deviation. The both‐wave subsample (*n* = 332) comprises participants who completed both T1 and T2 assessments and is therefore included within the T1 (*n* = 1061) and T2 (*n* = 812) columns; the three columns do not represent mutually exclusive groups. Accordingly, the total number of unique participants was 1541 (1061 + 812 − 332), which constitutes the analytic sample for the cross‐lagged models estimated with full information maximum likelihood (see Tables 3, 4, 5).

To examine the missing data mechanism, demographic characteristics were compared across the T1 sample, the T2 sample, and the both‐wave subsample. No significant differences emerged in gender (χ^2^(2) = 0.43, *p* = 0.808), age (*F*(2, 2202) = 2.01, *p* = 0.134), teaching experience (χ^2^(8) = 14.00, *p* = 0.082), or teaching subject (χ^2^(8) = 3.57, *p* = 0.894). However, differences were observed in school ownership (χ^2^(2) = 10.47, *p* = 0.005) and school type (χ^2^(8) = 52.70, *p* < 0.001), with teachers from public schools and junior vocational schools somewhat overrepresented, and senior high school teachers underrepresented, in the both‐wave subsample. Classification tests further indicated that T1 variables significantly predicted T2 missingness and vice versa (all *p*s ≤ 0.008). Little's MCAR test yielded χ^2^(32) = 46.36, *p* = 0.048. Taken together, these results suggest that the MCAR assumption is unlikely to hold. Because missingness was systematically related to observed variables, the pattern is consistent with, although it does not establish, a missing‐at‐random (MAR) mechanism. Under MAR, full information maximum likelihood (FIML) estimation yields unbiased parameter estimates provided that the variables related to missingness are incorporated into the estimation (Enders 2010). Two sets of such variables were identified in the present data and were accommodated as follows. First, the study variables themselves (the three PNT dimensions and turnover intention) predicted subsequent missingness; because these variables constitute the cross‐lagged models, the observed scores of single‐wave participants are used directly by FIML in estimating all model parameters. Second, school ownership and school type differed across the wave‐participation groups. Because neither variable was hypothesized to predict change in the T2 outcomes, they were not entered as structural covariates; instead, they were incorporated into the FIML estimation as auxiliary variables (see Section 2.3), so that the missing‐data model drew on this information without altering the substantive model. However, because the MAR assumption cannot be empirically verified, a missing‐not‐at‐random (MNAR) mechanism cannot be ruled out. This possibility is acknowledged as a limitation, and the longitudinal findings should be interpreted with corresponding caution (see Limitations). Nonetheless, because FIML under MAR represents the most defensible approach given the observed missingness pattern, it was retained for the primary analyses. Accordingly, missing data were handled differently depending on the analytic procedure. Descriptive statistics and zero‐order correlations were computed using the both‐wave subsample (*n* = 332), whereas FIML estimation was employed for the structural equation models. Consequently, the cross‐lagged models were estimated using all available observations from participants at either wave (analytic *N* = 1541, comprising all unique participants across the 1061 T1 and 812 T2 respondents), rather than being restricted to the both‐wave subsample (see the Data Analysis section below).

### 2.2 Measures

Chinese versions of validated measures were used to assess teachers' PNT and turnover intention.

#### 2.2.1 Psychological Need Thwarting

Teachers' PNT was assessed using the Chinese version of the PNT Scale for Teachers (CPNTT; Chen et al. 2020). The CPNTT comprises three subscales measuring autonomy thwarting (e.g., “I feel that I am prevented from making decisions about how I teach”), competence thwarting (e.g., “Sometimes I say things that make me feel incompetent”), and relatedness thwarting (e.g., “I feel that others are jealous when I achieve success”). Each subscale contains four items rated on a 7‐point Likert scale (1 = *strongly disagree* to 7 = *strongly agree*), with higher scores indicating greater need thwarting. The scale was originally developed for athletes (Bartholomew et al. 2011) and subsequently adapted for educational contexts (Cuevas et al. 2015), demonstrating robust psychometric properties across multiple studies. In this study, McDonald's omega coefficients indicated good to excellent internal consistency across all subscales and time points: autonomy thwarting (T1: ω = 0.83, T2: ω = 0.84), competence thwarting (T1: ω = 0.85, T2: ω = 0.87), and relatedness thwarting (T1: ω = 0.92, T2: ω = 0.92).

#### 2.2.2 Turnover Intention

The Turnover Intention Instrument (Kelloway et al. 1999) was used to assess teachers' intention to leave their current school. This unidimensional scale consists of four items rated on a 5‐point Likert scale (1 = strongly disagree to 5 = strongly agree), with higher scores indicating stronger turnover intentions. A sample item is: “I don't plan to be in this school much longer.” The scale demonstrated excellent internal consistency at both time points (McDonald's ω = 0.92 at T1 and  0.90 at T2).

### 2.3 Data Analysis

Preliminary analyses included descriptive statistics and Pearson correlations to examine initial patterns among study variables. Under the structural equation modeling framework, cross‐lagged panel modeling (CLPM) was employed to examine the temporal associations between teachers' PNT and turnover intention, with gender and age included as control variables. Following recommended practices for longitudinal analysis (Newsom 2015), measurement invariance across time points was first established through sequential confirmatory factor analyses (CFA), testing configural, metric, and scalar invariance. FIML estimation was utilized to handle missing data under the MAR assumption (see Section 2.1). To incorporate the observed demographic predictors of wave participation into the missing‐data model, school ownership (0 = public, 1 = private) and school type (four dummy variables with primary school as the reference category) were included in all CLPMs as auxiliary variables using the saturated‐correlates approach (Enders 2010). In this approach, the auxiliary variables are allowed to correlate with all exogenous variables and with the residuals of all endogenous variables, so that they inform the estimation of the missing values without entering the structural regressions. The structural model is therefore identical to that of a conventional CLPM. Both variables are time‐invariant and were observed for all 1541 participants, satisfying the conditions for effective auxiliary variables (Collins et al. 2001). For transparency, estimates from the corresponding models without auxiliary variables are reported in Supplementary Table S1.

Model fit was evaluated using multiple indices: the Comparative Fit Index (CFI), Tucker‐Lewis Index (TLI), Root Mean Square Error of Approximation (RMSEA), and Standardized Root Mean Square Residual (SRMR). According to Hu and Bentler (1999), acceptable model fit is indicated by CFI and TLI values ≥ 0.90, RMSEA ≤ 0.08, and SRMR ≤ 0.08. Following established guidelines (Chen 2007), measurement invariance was assessed through systematic comparisons of increasingly restrictive models. Metric invariance was supported when ΔCFI >−0.01, ΔRMSEA ≤ 0.015, and ΔSRMR ≤ 0.030, while scalar invariance required ΔCFI >−0.01, ΔRMSEA ≤ 0.015, and ΔSRMR ≤ 0.010. These criteria ensure that the measurement properties remained consistent across time points, providing a robust foundation for examining cross‐lagged relationships.

## 3 Results

### 3.1 Preliminary Analysis: Descriptive Statistics and Pearson Correlations

Table 2 presents the means and standard deviations for the three dimensions of PNT (autonomy, competence, and relatedness) and turnover intention at both time points. Among the three dimensions, competence thwarting exhibited the highest mean scores at both T1 and T2. Mean turnover intention scores fell below the theoretical scale midpoint of 12 at both time points.

**TABLE 2:** Descriptive statistics and correlations for psychological need thwarting and turnover intention (*n* = 332).

	*M (SD)*	1	2	3	4	5	6	7	8
1. PNT‐A at T1	14.01 (5.58)	—							
2. PNT‐C at T1	15.46 (5.74)	0.64^***^	—						
3. PNT‐R at T1	9.12 (4.75)	0.43^***^	0.48^***^	—					
4. Turnover Intention at T1	8.13 (3.19)	0.17^*^	0.23^*^	0.31^***^	—				
5. PNT‐A at T2	13.15 (5.49)	0.49^***^	0.46^***^	0.26^***^	0.15^**^	—			
6. PNT‐C at T2	15.02 (5.71)	0.38^***^	0.58^***^	0.28^***^	0.24^***^	0.70^***^	—		
7. PNT‐R at T2	9.52 (4.83)	0.29^***^	0.33^***^	0.48^***^	0.27^***^	0.50^***^	0.54^***^	—	
8. Turnover Intention at T2	8.43 (3.02)	0.19^*^	0.21^***^	0.25^***^	0.47^***^	0.30^***^	0.34^***^	0.41^***^	—

*Note*. Statistics are based on participants who completed both waves (listwise deletion). ^*^ *p* < 0.05, ^**^ *p* < 0.01, ^***^ *p* < 0.001; PNT‐A = Psychological Need Thwarting‐Autonomy; PNT‐C = Psychological Need Thwarting‐Competence; PNT‐R = Psychological Need Thwarting‐Relatedness; T1 = Time 1; T2 = Time 2; *M* = mean; *SD* = standard deviation.

Zero‐order correlations revealed that all three dimensions of PNT were positively correlated with turnover intention at each time point. Among the concurrent associations, relatedness thwarting demonstrated the strongest correlations with turnover intention (*r* = 0.31 at T1 and *r* = 0.41 at T2), followed by competence and autonomy thwarting. Examination of cross‐lagged correlations indicated significant positive associations between T1 PNT and T2 turnover intention, with relatedness thwarting again showing the strongest prospective association. Similarly, T1 turnover intention was positively correlated with T2 PNT across all three dimensions: autonomy (*r* = 0.15), competence (*r* = 0.24), and relatedness (*r* = 0.27). These preliminary findings suggest bidirectional associations between PNT and turnover intention, warranting further examination through cross‐lagged panel analysis.

### 3.2 Longitudinal Invariance Examination of Psychological Need Thwarting and Turnover Intention Measures

Four separate CFAs, one for each of the three PNT dimensions and one for turnover intention, were conducted using maximum likelihood estimation with robust standard errors (MLR) to examine measurement invariance across the four‐month study period. Initial models demonstrated acceptable fit indices, supporting configural invariance and indicating that the factorial structure remained equivalent across time points.

Metric invariance was established by constraining factor loadings across T1 and T2, with fit indices remaining within acceptable thresholds. Scalar invariance was subsequently supported through additional constraints on item intercepts, with no significant deterioration in model fit. This confirmation of full scalar invariance for all constructs (see Table 3) provides a robust foundation for subsequent CLPM analyses.

**TABLE 3:** Longitudinal measurement invariance testing: psychological need thwarting scale for teachers (autonomy, competence, and relatedness) and turnover intention instrument (FIML).

Model	χ^2^	*df*	CFI	RMSEA	SRMR	Δχ^2^	Δ*df*	ΔCFI	ΔRMSEA	ΔSRMR
**Autonomy thwarting**										
Configural invariance	14.57	15	0.999	0.000	0.021	—	—	—	—	—
Metric invariance	17.09	18	0.999	0.000	0.024	2.52	3	0	0	0.003
Scalar invariance	27.61	21	0.999	0.014	0.026	10.52	3	0	0.014	0.002
**Competence thwarting**										
Configural invariance	15.79	15	0.999	0.006	0.023					
Metric invariance	21.18	18	0.999	0.011	0.030	5.39	3	0	0.005	0.007
Scalar invariance	31.19	21	0.996	0.018	0.032	10.01	3	−0.003	0.007	0.002
**Relatedness thwarting**										
Configural invariance	47.91	15	0.988	0.038	0.023					
Metric invariance	56.18	18	0.986	0.037	0.027	8.27	3	−0.002	−0.001	0.004
Scalar invariance	63.92	21	0.984	0.036	0.028	7.74	3	−0.002	−0.001	0.001
**Turnover Intention**										
Configural invariance	48.60	15	0.985	0.038	0.020					
Metric invariance	52.68	18	0.985	0.035	0.021	4.08	3	0	−0.003	0.001
Scalar invariance	55.19	21	0.985	0.033	0.022	2.51	3	0	−0.002	0.001

*Note*. *N* = 1541 unique participants contributed data to at least one wave; *n* = 332 completed both waves. Models were estimated with full information maximum likelihood (FIML) using all available observations. χ^2^ = chi‐square statistic; df = degrees of freedom; CFI = comparative fit index; RMSEA = root mean square error of approximation; SRMR = standardized root mean square residual.

### 3.3 Cross‐Lagged Panel Models

Table 4 presents three CLPMs, all including gender and age as control variables and school ownership and school type as auxiliary variables, with acceptable model fit indices supporting the examination of path coefficients. The cross‐lagged paths (unstandardized coefficients with 95% confidence intervals in Table 5, standardized in Figures 1, 2, 3) revealed differential directional relationships between PNT dimensions and turnover intention.

**TABLE 4:** Model fit indices for cross‐lagged panel models (FIML).

	*χ^2^* (*df*)	CFI	TLI	RMSEA (90 percent confidence interval); RMSEA *p*‐value	SRMR
The association of autonomy thwarting with turnover intention	160.56 (114)	0.994	0.992	0.016 (0.010–0.022); 0.999	0.020
The association of competence thwarting with turnover intention	330.79 (114)	0.980	0.973	0.035 (0.031–0.039); 0.999	0.053
The association of relatedness thwarting with turnover intention	240.93 (114)	0.988	0.983	0.027 (0.023–0.031); 0.999	0.020

*Note. N* = 1541 unique participants contributed data to at least one wave; *n* = 332 completed both waves. Models were estimated with full information maximum likelihood (FIML) using all available observations, with school ownership and school type included as auxiliary variables (saturated‐correlates approach). χ^2^ = chi‐square statistic; df = degrees of freedom; CFI = comparative fit index; TLI = Tucker–Lewis index; RMSEA = root mean square error of approximation; SRMR = standardized root mean square residual.

**TABLE 5:** Summary of the cross‐lagged panel models (FIML).

	Covariance/coefficient	*p*
**Covariance between variables on each occasion**		
Autonomy thwarting and turnover intention at T1	0.22 (0.16, 0.29)	<0.001
Autonomy thwarting and turnover intention at T2	0.14 (0.06, 0.21)	<0.001
Competence thwarting and turnover intention at T1	0.31 (0.22, 0.40)	<0.001
Competence thwarting and turnover intention at T2	0.17 (0.06, 0.28)	0.002
Relatedness thwarting and turnover intention at T1	0.39 (0.31, 0.47)	<0.001
Relatedness thwarting and turnover intention at T2	0.21 (0.12, 0.30)	<0.001
**Autoregression paths**		
Autonomy thwarting T1 to autonomy thwarting T2	0.59 (0.45, 0.72)	<0.001
Competence thwarting T1 to competence thwarting T2	0.58 (0.49, 0.68)	<0.001
Relatedness thwarting T1 to relatedness thwarting T2	0.45 (0.33, 0.57)	<0.001
Turnover intention T1 to turnover intention T2: autonomy thwarting model	0.51 (0.39, 0.62)	<0.001
Turnover intention T1 to turnover intention T2: competence thwarting model	0.51 (0.40, 0.63)	<0.001
Turnover intention T1 to turnover intention T2: relatedness thwarting model	0.50 (0.38, 0.63)	<0.001
**Cross‐lagged paths**		
Autonomy thwarting T1 to turnover intention T2	0.09 (0.01, 0.17)	0.023
Turnover intention T1 to autonomy thwarting T2	0.08 (‐0.07, 0.24)	0.276
Competence thwarting T1 to turnover intention T2	0.04 (0.00, 0.09)	0.056
Turnover intention T1 to competence thwarting T2	0.22 (0.02, 0.41)	0.031
Relatedness thwarting T1 to turnover intention T2	0.05 (‐0.03, 0.13)	0.214
Turnover intention T1 to relatedness thwarting T2	0.19 (0.01, 0.37)	0.048

*Note. N* = 1541 unique participants contributed data to at least one wave; *n* = 332 completed both waves. Models were estimated with full information maximum likelihood (FIML) using all available observations, with school ownership and school type included as auxiliary variables (saturated‐correlates approach). Gender and age were included as control variables. T1 = Time 1; T2 = Time 2.

**FIGURE 1:**
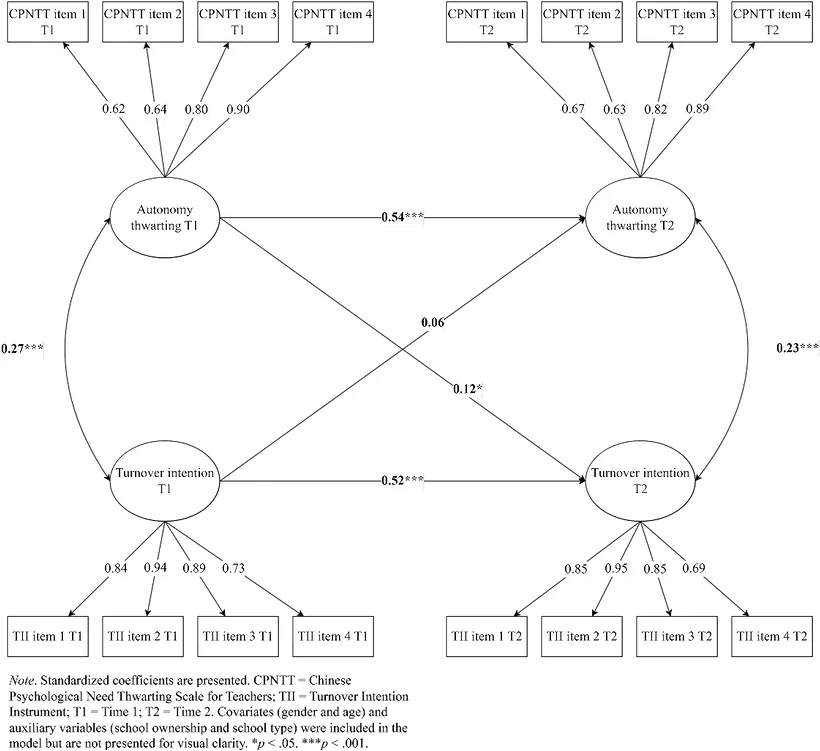
Cross‐lagged panel model for the bidirectional relationships between autonomy thwarting and turnover intention with standardized path coefficients.

**FIGURE 2:**
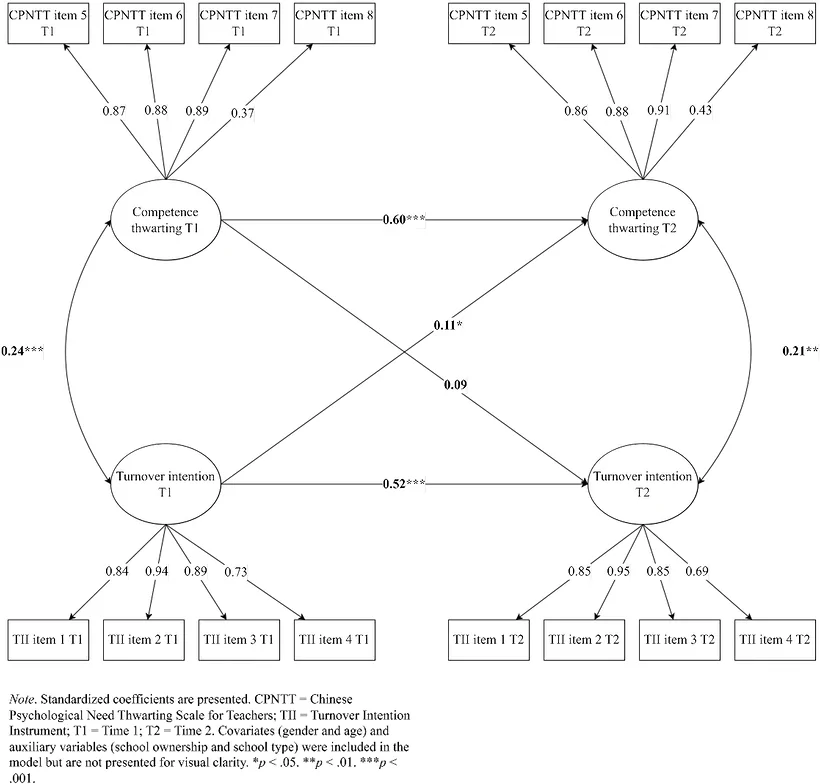
Cross‐lagged panel model for the bidirectional relationships between competence thwarting and turnover intention with standardized path coefficients.

**FIGURE 3:**
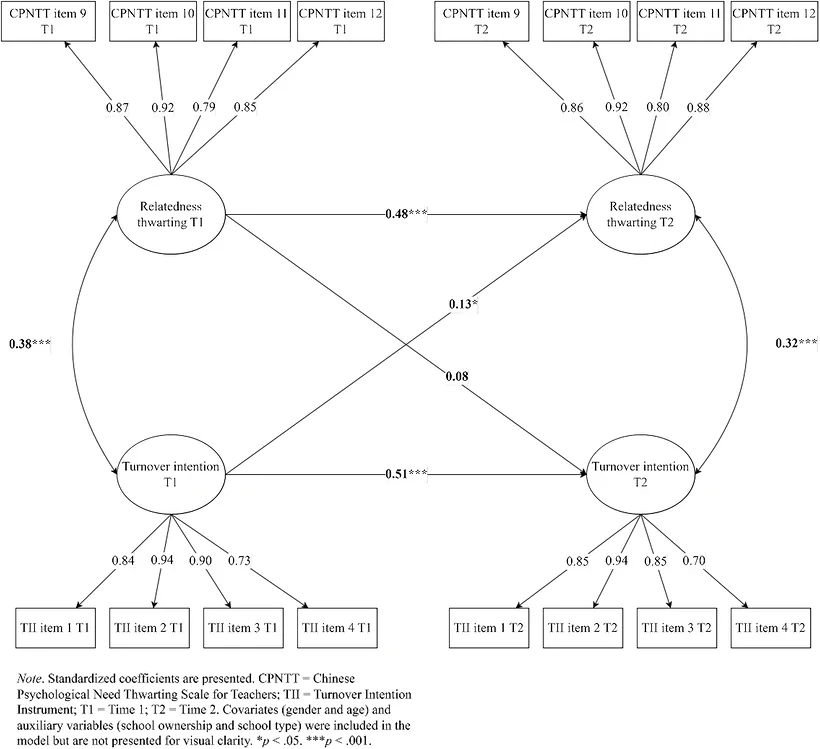
Cross‐lagged panel model for the bidirectional relationships between relatedness thwarting and turnover intention with standardized path coefficients.

Autonomy thwarting at T1 significantly predicted turnover intention at T2 (B = 0.09, 95% CI [0.01, 0.17], β = 0.12, *p* = 0.023), though the reverse path was non‐significant (B = 0.08, 95% CI [−0.07, 0.24], β = 0.06, *p* = 0.276). Conversely, turnover intention at T1 predicted increased competence thwarting (B = 0.22, 95% CI [0.02, 0.41], β = 0.11, *p* = 0.031) and relatedness thwarting (B = 0.19, 95% CI [0.01, 0.37], β = 0.13, *p* = 0.048) at T2, whereas the corresponding forward paths from competence thwarting (B = 0.04, 95% CI [0.00, 0.09], β = 0.09, *p* = 0.056) and relatedness thwarting (B = 0.05, 95% CI [−0.03, 0.13], β = 0.08, *p* = 0.214) to later turnover intention did not reach significance. Additionally, autoregressive paths demonstrated strong temporal stability for all constructs, with standardized coefficients ranging from 0.48 to 0.60 (all *p*s < 0.001). Neither gender nor age significantly predicted the T2 variables in any model (all *p*s > 0.05). Estimates from the same models without the auxiliary variables (Supplementary Table S1) were essentially identical: no standardized cross‐lagged coefficient differed by more than 0.02, and no path changed in statistical significance, indicating that the findings are not sensitive to the inclusion of the observed demographic predictors of wave participation in the missing‐data model.

## 4 Discussion

Although PNT has been consistently identified as being associated with teachers' turnover intention (e.g., Collie 2023; Shim et al. 2022), most prior studies employed cross‐sectional designs, treated PNT as a unidimensional construct, and focused primarily on the pathway from PNT to turnover intention without considering potential reverse pathways. This study addressed these limitations by using a two‐wave CLPM to examine reciprocal temporal associations between specific PNT dimensions and turnover intention. The results partially supported the hypotheses, revealing distinct temporal patterns and suggesting that the three dimensions of PNT may function differently in relation to turnover intention.

### 4.1 From Psychological Need Thwarting to Turnover Intention

The findings indicated that only autonomy thwarting at the initial time point was significantly associated with subsequent turnover intention, whereas competence and relatedness thwarting showed no significant prospective associations. These results partially supported Hypothesis 1, corroborating the salient role of autonomy thwarting in relation to turnover intention, yet diverging from prior studies in which multiple need dimensions were associated with turnover intention. For instance, Shim et al. (2022) found that both autonomy and relatedness thwarting were positively associated with turnover intention among European American teachers. Similarly, using latent profile analysis among Finnish teachers, Heinimäki et al. (2026) found that profiles characterized by elevated relatedness thwarting or competence thwarting also reported higher turnover intention. These cross‐sectional findings suggest that all three dimensions of thwarting may be linked to turnover intention; however, the temporal precedence of these associations awaits further confirmation.

A plausible explanation for these discrepancies lies in methodological variation. Prior research (e.g., Heinimäki et al. 2026; Shim et al. 2022) relied on cross‐sectional methods, which are limited in accounting for the fluctuations and changes of psychological variables within dynamic environments. In contrast, the CLPM employed in this study controlled for autoregressive effects (temporal stability) of the variables (Selig and Little 2012), focusing specifically on their prospective associations. Overall, the present findings suggest that autonomy thwarting temporally relates to turnover intention among Chinese teachers. This is consistent with Chirkov et al. (2003), who demonstrated that autonomy remains functionally important in collectivist contexts, challenging earlier cross‐cultural assumptions that treated autonomy as primarily a Western, individualist concern.

Notably, although competence and relatedness thwarting were concurrently associated with turnover intention, they did not show significant temporal associations with later turnover intention. One possible explanation for this differential pattern centers on the unique relationship between autonomy and turnover intention. Turnover intention is fundamentally a volitional construct: it concerns the *choice* to stay or leave an organization. Among the three basic psychological needs, autonomy is the need most directly connected to volition, concerning one's sense of agency, self‐direction, and ownership over decisions (Deci and Ryan 2000; Ryan and Deci 2017). When autonomy is thwarted, teachers may experience a loss of control over their professional lives, feel coerced by external demands, and perceive their actions as externally dictated rather than self‐endorsed. In such circumstances, forming an intention to leave may represent a psychologically coherent response, reflecting a possible attempt to reclaim volitional control by exiting the constraining environment. The conceptual alignment between autonomy, as volition over one's actions, and turnover intention, as volition to exit, may help explain why only autonomy thwarting showed a prospective association with subsequent turnover intention.

In contrast, competence and relatedness thwarting, while distressing, may not be directly aligned with the volitional core that underlies turnover intention. One possible interpretation is that these forms of thwarting may elicit adaptive coping responses that allow teachers to manage distress while remaining within the organization. Research on job crafting from the SDT perspective indicates that employees can proactively shape their work environments to satisfy their psychological needs (Slemp and Vella‐Brodrick 2014). When teachers experience competence thwarting, they may respond through cognitive crafting strategies, such as lowering performance expectations, seeking additional professional development, or redefining their standards of success, that do not require organizational exit. When relatedness thwarting occurs, teachers may compensate by seeking meaningful connections outside the workplace, emotionally distancing themselves from colleagues, or accepting social isolation as a tolerable aspect of professional life. Furthermore, contextual factors may constrain the behavioral options available to competence‐thwarted teachers. Classic turnover theory posits that employees' decisions to leave depend not only on job dissatisfaction but also on perceived ease of movement and job alternatives (Mobley et al. 1979). Teachers who feel ineffective at their current job may perceive limited prospects in a competitive external labor market, reducing the viability of exit as a coping strategy. In sum, while competence and relatedness thwarting may contribute to dissatisfaction and disengagement, the present findings suggest that they may not engage the volitional foundation of staying or leaving, and thus may not translate into turnover intention over time. These interpretations remain tentative, however, because coping strategies, job crafting behaviors, and perceived job alternatives were not directly measured in the present study.

### 4.2 From Turnover Intention to Psychological Need Thwarting

The findings only partially supported Hypothesis 2: turnover intention at the initial time point was prospectively associated with subsequent competence and relatedness thwarting, but not autonomy thwarting. Extending previous research that has largely overlooked this possibility, these results provide preliminary longitudinal evidence for a reverse temporal association. Drawing on COR theory (Hobfoll et al. 2018), one possible interpretation is that elevated turnover intention may reflect or coincide with a state of resource strain, which could be accompanied by defensive and withdrawal‐oriented coping strategies aimed at conserving remaining resources. Such strategies may include reduced work engagement, avoidance of professional challenges, and social disengagement from colleagues. Over time, these withdrawal behaviors may be linked to lower teaching effectiveness and weakened interpersonal relationships, which may be followed by increased competence and relatedness thwarting through a loss spiral.

However, turnover intention was not significantly associated with subsequent autonomy thwarting. Several explanations may account for this null finding. One possibility draws on psychological reactance theory (Brehm 1966; Brehm and Brehm 1981), which posits that perceived threats to behavioral freedom may elicit a motivational state aimed at restoring that freedom. From this perspective, forming an intention to leave may function as a cognitive means of reasserting volitional control: by affirming the possibility of exit, teachers may preserve their subjective sense of autonomy even when objective working conditions remain unchanged. In this sense, turnover intention may not necessarily be followed by increased autonomy thwarting.

An alternative explanation centers on the structural nature of autonomy thwarting. Unlike competence and relatedness, which may be more closely shaped by interpersonal dynamics and individual behaviors, autonomy thwarting may be more firmly rooted in organizational structures such as institutional policies, supervisory practices, and decision‐making hierarchies. These structural factors may be relatively impervious to individual teachers' psychological states or withdrawal behaviors, potentially explaining why turnover intention showed no prospective association with autonomy thwarting, in contrast to the associations observed for competence and relatedness thwarting.

These two explanations are not mutually exclusive, and future research employing more fine‐grained assessments of both cognitive appraisals and organizational conditions would help clarify the mechanisms underlying this asymmetric pattern.

### 4.3 Theoretical and Practical Implications

The present findings contribute to the SDT literature by demonstrating that the three dimensions of PNT do not function uniformly in relation to turnover intention. Rather than treating it as a unidimensional construct, the current study provides evidence of dimension‐specific temporal dynamics: only autonomy thwarting predicted subsequent turnover intention, whereas competence and relatedness thwarting did not. This pattern is consistent with the theoretical proposition that autonomy holds a privileged position among the basic psychological needs due to its direct connection to volition and self‐regulation (Ryan and Deci 2017). The study also extends the turnover literature by providing preliminary longitudinal evidence for a reverse pathway from turnover intention to PNT. Whereas existing models predominantly position turnover intention as an outcome of workplace experiences, the present findings suggest that turnover intention may also be prospectively associated with subsequent need experiences. By integrating COR theory (Hobfoll et al. 2018) and psychological reactance theory (Brehm and Brehm 1981), this study offers a possible theoretical account of why turnover intention may be differentially related to the three need dimensions: withdrawal behaviors associated with turnover intention may be linked to later competence and relatedness thwarting, whereas the volitional nature of turnover intention may help preserve subjective autonomy. Collectively, these findings point to the value of reconceptualizing turnover models from static, unidirectional frameworks to dynamic, reciprocal processes.

The differential temporal patterns observed in this study suggest possible directions for tailoring intervention strategies to teachers' current psychological states. For teachers who have not yet developed turnover intention, prevention efforts may benefit from prioritizing autonomy support, given that autonomy thwarting showed the clearest prospective association with subsequent turnover intention. Educational administrators can foster autonomy by granting teachers greater instructional discretion, involving them in decision‐making processes, providing meaningful rationales for institutional policies, and minimizing controlling supervisory practices (Slemp et al. 2021). For teachers who already exhibit elevated turnover intention, intervention efforts may need to consider competence and relatedness support, given the observed associations between turnover intention and later competence and relatedness thwarting. Competence support may involve constructive performance feedback and structured opportunities for professional development (Slemp et al. 2021), while relatedness support may involve fostering collaborative activities such as team teaching and peer learning communities that promote genuine interpersonal connection (Vangrieken et al. 2015). It should be emphasized, however, that the cross‐lagged coefficients observed here were small in magnitude (β = 0.11–0.13), with several approaching the conventional significance threshold, and that no intervention was tested in the present design. These implications are therefore advanced as hypotheses and promising targets for future experimental or multi‐wave longitudinal research, rather than as established intervention effects.

### 4.4 Limitations and Future Directions

Several limitations of this study warrant attention. First, all data were collected via self‐report measures, which may introduce common method bias and social desirability effects. This concern is particularly relevant because turnover intention is a sensitive work‐related attitude, and teachers may underreport it due to professional norms or job‐security concerns. In addition, the exclusion criterion regarding clinical mental disorders relied on a self‐report screening item and was not clinically verified. Future research may benefit from incorporating objective indicators or multi‐source assessments.

Second, the study focused exclusively on the direct relationships between PNT and turnover intention without examining potential mediators (e.g., burnout, job satisfaction) or moderators (e.g., organizational support, career stage) that may clarify the underlying mechanisms. Future studies should extend the current model by incorporating such variables.

Third, the two‐wave design, while advancing beyond cross‐sectional approaches, remains insufficient for capturing complex developmental trajectories. As a result, it is difficult to determine whether the observed associations reflect stable longitudinal patterns, short‐term fluctuations, or longer‐term developmental processes. Moreover, this design may conflate between‐person differences with within‐person changes, because it cannot fully separate stable individual differences from changes occurring within the same individual over time. Future research should employ more measurement occasions and sophisticated analytical techniques such as the Random Intercept Cross‐Lagged Panel Model.

Fourth, the four‐month interval between waves may not optimally capture the temporal dynamics of need thwarting and turnover intention, and alternative intervals should be explored in future work. Fifth, although FIML estimation was employed to address missing data under an assumption of missingness at random, this assumption is supported by, but not established by, the observed classification tests. In particular, teachers with stronger turnover intentions at T1, including those who had actually left their positions before T2, may have been less likely to participate at the follow‐up. Such selective non‐participation would constitute a missing‐not‐at‐random mechanism, and the present data cannot exclude this possibility. Moreover, teachers from private schools and senior high schools were underrepresented among both‐wave completers; although these characteristics were incorporated into the FIML estimation as auxiliary variables, and the estimates proved insensitive to their inclusion, the generalizability of the longitudinal findings to these groups remains uncertain. Sixth, the study measured turnover intention rather than actual turnover behavior, and future research should examine whether the observed patterns extend to behavioral outcomes.

## 5 Conclusion

Although PNT has been identified as a predictor of teachers' turnover intention, prior research has predominantly relied on cross‐sectional designs, treated need thwarting as a unidimensional construct, and overlooked potential reverse pathways. By employing a two‐wave CLPM, the present study observed an asymmetric temporal pattern: among the three dimensions of need thwarting, only autonomy thwarting was associated with subsequent turnover intention, while turnover intention was associated with subsequent competence and relatedness thwarting but not autonomy thwarting. This asymmetry aligns with theoretical expectations but should be interpreted cautiously given the observational design, and the potential mechanisms underlying it, as discussed above, remain to be directly tested. These findings suggest that potentially relevant intervention targets may need to be differentiated: autonomy support may help reduce the emergence of turnover intention, whereas competence and relatedness support may be particularly relevant for teachers already contemplating departure. Overall, turnover intention may not merely be an endpoint of adverse workplace experiences but may also function as a psychological state associated with changes in how teachers experience their professional environment.

## Author Contributions

**I‐Hua Chen**: conceptualization, methodology, supervision, formal analysis and investigation. **Qi Song and I‐Hua Chen**: writing – original draft preparation, writing – review and editing. **I‐Hua Chen, Xiao‐Yan Zou, Cong Liu, and Shan Xu**: resources. All authors have read and agreed to the published version of the manuscript.

## Funding

This work was supported by the Philosophy and Social Science Research Project of the Department of Education of Hubei Province (Project No. 25ZX136).

## Ethics Statement

The research protocol received full ethical approval from the Institutional Review Board of the Jiangxi Psychological Consultant Association (IRB Reference: JXSXL‐2024‐JA08). All procedures were conducted in strict accordance with established ethical guidelines for research involving human participants.

## Supporting information




**Supplementary Table**: brb371861‐sup‐0001‐TableS1.docx

## Data Availability

The data are not publicly available due to privacy and ethical restrictions.
