# Supplementary material for: Not All Thwarting Is Equal: Distinct Temporal Patterns Linking Teachers' Psychological Needs to Turnover Intention
Source: Brain Behav. 2026 Oct 28;16(11):e71861. doi: 10.1002/brb3.71861 (PMC13715077; doi:10.1002/brb3.71861)
Supplement: Supplementary file 1 [file BRB3-16-e71861-s001.docx]

**Table S1.** Cross-Lagged Paths From the Cross-Lagged Panel Models Estimated With and Without School Ownership and School Type as Auxiliary Variables

| **Cross-lagged path** | **Without auxiliary variables** | | | | **With auxiliary variables**  **(reported in Tables 4–5)** | | | |
| --- | --- | --- | --- | --- | --- | --- | --- | --- |
|  | ***B*** | **95% CI** | ***β*** | ***p*** | ***B*** | **95% CI** | ***β*** | ***p*** |
| T1 Autonomy thwarting → T2 Turnover intention | 0.10 | [0.02, 0.18] | 0.12 | .015 | 0.09 | [0.01, 0.17] | 0.12 | .023 |
| T1 Turnover intention → T2 Autonomy thwarting | 0.09 | [−0.06, 0.24] | 0.07 | .246 | 0.08 | [−0.07, 0.24] | 0.06 | .276 |
| T1 Competence thwarting → T2 Turnover intention | 0.04 | [−0.00, 0.09] | 0.09 | .058 | 0.04 | [−0.00, 0.09] | 0.09 | .056 |
| T1 Turnover intention → T2 Competence thwarting | 0.21 | [0.01, 0.40] | 0.11 | .035 | 0.22 | [0.02, 0.41] | 0.11 | .031 |
| T1 Relatedness thwarting → T2 Turnover intention | 0.06 | [−0.02, 0.14] | 0.10 | .130 | 0.05 | [−0.03, 0.13] | 0.08 | .214 |
| T1 Turnover intention → T2 Relatedness thwarting | 0.19 | [0.00, 0.37] | 0.13 | .047 | 0.19 | [0.00, 0.37] | 0.13 | .048 |

*Note. N* = 1,541 unique participants contributed data to at least one wave; *n* = 332 completed both waves. All models included gender and age as control variables and were estimated with full information maximum likelihood (FIML) and robust maximum likelihood (MLR) in R. In the models with auxiliary variables, school ownership (0 = public, 1 = private) and school type (four dummy variables; reference = primary school) were incorporated using the saturated-correlates approach; the auxiliary variables do not enter the structural regressions, so the structural model is identical in both sets of models. B = unstandardized coefficient; CI = confidence interval; β = standardized coefficient.
